# A Critical Review on the Use of Ionic Liquids in Proton Exchange Membrane Fuel Cells

**DOI:** 10.3390/membranes12020178

**Published:** 2022-02-02

**Authors:** Adnan Alashkar, Amani Al-Othman, Muhammad Tawalbeh, Muhammad Qasim

**Affiliations:** 1 Materials Science and Engineering Ph.D. Program, Department of Chemical Engineering, American University of Sharjah, Sharjah P.O. Box 26666, United Arab Emirates; b00028197@alumni.aus.edu; 2 Department of Chemical Engineering, American University of Sharjah, Sharjah P.O. Box 26666, United Arab Emirates; mqasim@aus.edu; 3 Department of Sustainable and Renewable Energy Engineering, University of Sharjah, Sharjah P.O. Box 27272, United Arab Emirates; mtawalbeh@sharjah.ac.ae; 4 Sustainable Energy & Power Systems Research Centre, Research Institute of Sciences & Engineering (RISE), University of Sharjah, Sharjah P.O. Box 27272, United Arab Emirates

**Keywords:** proton exchange membrane, fuel cells, ionic liquids, polymers, Nafion membrane

## Abstract

This work provides a comprehensive review on the incorporation of ionic liquid (ILs) into polymer blends and their utilization as proton exchanges membranes (PEM). Various conventional polymers that incorporate ILs are discussed, such as Nafion, poly (vinylidene fluoride), polybenzimidazole, sulfonated poly (ether ether ketone), and sulfonated polyimide. The methods of synthesis of IL/polymer composite membranes are summarized and the role of ionic liquids as electrolytes and structure directing agents in PEM fuel cells (PEMFCs) is presented. In addition, the obstacles that are reported to impede the development of commercial polymerized IL membranes are highlighted in this work. The paper concludes that the presence of certain ILs can increase the conductivity of the PEM, and consequently, enhance the performance of PEMFCs. Nevertheless, the leakage of ILs from composite membranes as well as the limited long-term thermal and mechanical stability are considered as the main challenges that limit the employment of IL/polymer composite membranes in PEMFCs, especially for high-temperature applications.

## 1. Introduction

Over recent decades, renewable energy systems have been utilized to meet the increasing demand for power and energy. Among these renewable energy systems, fuel cells seem to be receiving extra attention. A fuel cell produces electricity by utilizing hydrogen or other fuels via an electrochemical reaction. Fuel cells are suitable for stationary and portable applications. In addition, they serve as a potential candidate for transportation due to their high power density, low operating temperature, and dynamic characteristics [1]. Fuel cells operate at a higher efficiency compared to combustion engines [2]. Fuel cells have been reported to convert chemical energy to electricity with a theoretical efficiency of about 83% for hydrogen fuel cells specifically [3]. This efficiency is defined based on the ratio of the amount of Gibbs free energy divided by the enthalpy or the heat of combustion for the overall reaction in a hydrogen fuel cell. As a result, fuel cells offer several advantages when compared to conventional combustion engines. In addition to superior efficiency, fuel cells have less carbon dioxide and greenhouse gas (GHG) emissions compared to combustion engines [4]. In fact, during the operation of hydrogen fuel cells, there are no carbon dioxide or air pollutants emissions. Another advantage of fuel cells is their silent operation, as they possess fewer dynamic parts when compared to combustion engines.

Proton-exchange membrane fuel cells (PEMFCs) have received considerable research interest recently. PEMFCs have an all-solid structure that makes them ideal for transport applications, stationary, and portable applications. These fuel cells utilize a special polymer electrolyte membrane for conducting protons. PEMFCs can produce clean energy while operating at temperatures lower than other types of fuel cells. PEMFCs are also distinguished by their silent operation and all-solid membrane structure due to the polymer electrolyte. Lately, research has been conducted to enhance their durability, cost, and overall performance. However, improvements in the technical and economical aspects of the fuel cell are still required for commercialization. Research has been focusing on the optimization of the fuel cell by optimizing the configuration, materials, conformation, and structure of the catalyst layer [5,6,7,8,9]. For instance, several research studies have focused on decreasing the cost of PEMFCs by enhancing the electrolyte conductivity [10,11,12,13]. Simultaneously, in order to enhance the safety of the fuel cells, nonflammable and nonvolatile electrolytes have been investigated.

PEMFCs have been investigated for their application in electric vehicles. Although the industry is predominantly reliant on lithium-ion batteries, PEMFCs have great potential in powering and improving the performance of electric vehicles [14]. Moreover, PEMFCs are able to tap into the market and compete with lithium-ion batteries to offer electric vehicles that have a long driving range, high utilization, and low cost [14,15]. The main advantage of PEMFCs over batteries, especially in the long-range market, is the high specific energy and energy density of hydrogen [16]. In addition, for future high-utilization transportation, hydrogen is a natural fit when looking at grid compatibility and fast refueling.

Ionic liquids (ILs) possess desired characteristics such as excellent ionic conductivity even at anhydrous conditions [17]. Many research efforts have been conducted on the incorporation of ILs into PEMFCs, yet, review articles pertaining to the subject are either outdated or focused only on certain types of polymers or ILs [18,19]. This work provides a comprehensive review on the recent use of ILs in PEMFCs, focusing mainly on systems employing IL/polymer composite membranes while outlining the future research needs and challenges. This review has also presented the opportunities for the use of ILs in high-temperature operations and focused on presenting all types of polymers evaluated in the literature for this purpose.

## 2. Proton Exchange Membrane Fuel Cells (PEMFCs) 

**Figure 1:**
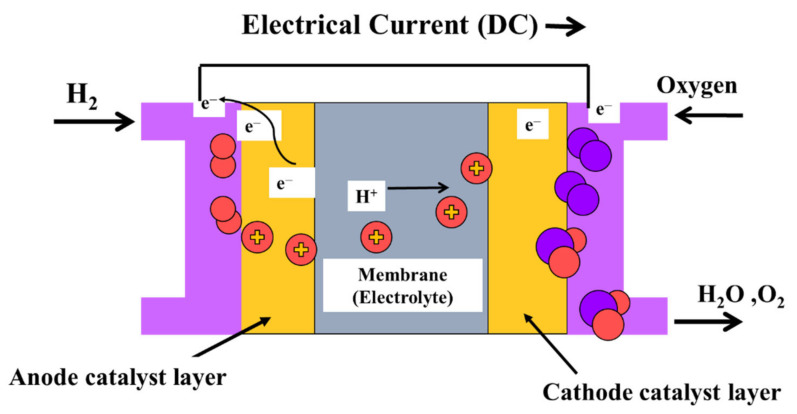
Schematic diagram of a PEMFC.

A PEMFC consists of electrodes, an electrolyte, a catalyst, and diffusion layers. Figure 1 depicts a diagram of a PEMFC [18]. The membrane and the catalyst layers create the membrane electrode assembly. The PEM serves a vital role, as it is responsible for separating the gaseous reactants, conducting protons, and electrically insulating the electrons. Membranes ideal for PEMFCs should be of low cost and possess desirable properties such as high proton conductivity, high mechanical strength, high thermal, chemical, and electrochemical stability, and low oxygen and fuel crossover. In that regard, perfluorosulfonic acid (PFSA) membrane, i.e., Nafion of Dupont, is most commonly in PEMFCs, due to its superb chemical stability, outstanding strength, and high ionic conductivity [20,21]. Nevertheless, the conductivity of the membrane decreases significantly when the temperature exceeds 100 °C, rendering it undesirable for high-temperature processes.

Proton exchange membranes could be classified based on their properties and functions. The structure of the PFSA membranes consists of a fluorinated backbone with a fluorocarbon side chain, in addition to sulphonic acid ion clusters attached to the side chains of the backbone. These membranes are prone to dehydration at temperatures above 80 °C, which, consequently, reduces their proton conductivity. In order to overcome the challenges faced by PFSA membranes, researchers have adopted partially fluorinated membranes [22]. The structure of the partially fluorinated membranes consists of a fluorocarbon base with a hydrocarbon side chain grafted on the backbone. Hence, the structure allows for the use of inexpensive base films with a low crossover, in addition to forming a relatively stronger compound compared to PFSA membranes. Moreover, the cost of the membrane is reduced since a less fluorinated polymer is needed; nevertheless, the reduction in the cost comprises the proton conductivity resulting in low performance and less durable membrane compared to a perfluorinated polymer.

Researchers have studied the use of nonfluorinated membranes as alternatives to conventional PFSA membranes in PEMFCs. The nonfluorinated membranes consist of a hydrocarbon base, normally altered by the addition of polar or sulphonic groups. The polymer membranes should have a proton conductor component. Research in this context focused first on poly (arylene ether) materials, due to their high stability, availability, and processibility [23]. Nevertheless, due to the short lifetime and excessive swelling of poly (arylene ether) membranes, efforts were directed towards optimizing other nonfluorinated materials and acid–base membranes. The latter involves the incorporation of acid components (such as sulfonated compounds) into an alkaline polymer base such as polybenzimidazole (PBI). The mixture of the acid–base membrane allows for high thermal and chemical stabilities, in addition to a proton conductivity in the same range as, or even higher than, that of Nafion. Among the strong acids, phosphoric acid (H_3_PO_4_) and sulphuric acid (H_2_SO_4_) show valuable proton conductivities in both wet and anhydrous forms [24,25]. PBI is known for its high chemical and thermal stability. However, the main disadvantage of the acid–base membrane is the leaching of acid during operation, which limits the durability of the membrane [18]. Table 1 provides a summary of the classification of membranes in terms of their category and structure. A detailed summary of the structure, physical properties, and performance of membranes can be found in [26].

Significant research has been performed on developing novel proton exchange membranes for use in fuel cells [12,13,27]. These efforts have achieved moderate success. However, it is clear that these membranes still need improvement to become commercially viable. Researchers have investigated the modification of Nafion membranes by incorporating bifunctional compounds, such as silica, zeolites, and carbon nanotubes, to enhance water retention and increase proton conductivity at higher temperatures [28]. 

The electrodes of the proton exchange membrane represent another vital component in PEMFCs arrangement. In order to overcome the challenges halting the utilization of PEMFCs as an efficient and competitive energy source, the performance and durability of the electrolyte and electrodes need to be improved. Typically, the electrodes consist of three layers: backing, gas diffusion, and catalyst layers [29]. The catalyst layer usually consists of noble metals, such as platinum, as a catalyst; however, platinum is quite expensive and accounts for 55% of the PEMFC cost. In order to lower the cost without significantly compromising the performance of PEMFCs, platinum alloys (such as Pt–Ru, Pt–Co, Pt–Pd, etc.) are employed as catalysts [29]. Another method is the use of platinum bimetal, which involves adding metals such as Fe, Cu, Co, and Ni to the platinum catalyst. Moreover, the catalyst ink distribution and the agglomeration of catalyst particles are crucial to the performance of the catalyst layer.

In the subsequent sections, the focus will be on discussing the recent developments in the incorporation of ILs in PEMFCs polymer membranes.

## 3. Ionic Liquids Applications in PEM

ILs are salts in the liquid state, typically defined as liquid electrolytes composed entirely of ions without the use of solvents. Some ILs flow freely as liquids at room temperature. These ILs are referred to as room-temperature ILs (RTILs). Furthermore, ILs possess a number of desirable properties for electrochemical applications, such as: very low volatility, nonflammable characteristics, high thermal and electrochemical stability, and high ionic conductivity under anhydrous conditions [19]. Given the excellent properties of ILs, and the fact that they are made up entirely from ions and do not contain solvents, ILs can be combined and/or modified to tailor their properties that are desirable for specific applications. For instance, extensive research has been carried for the employment of IL as electrolytes in solar cells, batteries, and fuel cells [30,31,32].

The critical aspect of the fuel-cell performance is stability, which is heavily impacted by the conductivity of the IL. Typically, ILs possess conductivities that are in the range 1.0 × 10^−4^–1.8 × 10^−4^ S/cm [19]. For instance, ILs based on imidazolium cations exhibit conductivity of approximately 1 mS/cm [19]. Other ILs, such as those based on pyrrolidinium, piperidinium, pyridinium, and tetraalkylammonium cations, show lower conductivities (in the range 1.0 × 10^−4^–5 × 10^−3^ S/cm). The counter ion influences the reduction potential of the cation or the anion. It has been reported that the presence of halide anions, such as F^−^ and Br^−^, limits the stability to 2–3 V. However, in the case of the bis(trifluoromethylsulphonyl)imide (TFSI^−^) anion, higher stability in the region of 4.5 V could be obtained due to the oxidization of the anion at a high anodic potential [33]. ILs with tetraalkylammonium cations display improved stability in the range of 4.0–5.7 V due to the cathodic reduction occurring at temperately negative potentials [34].

ILs can be either protic or aprotic. Protic ILs are suitable for application in fuel cells due to the reactivity of the active proton available at the cation. On the other hand, aprotic ILs, i.e., not protic, possess low melting points owing to the padding of large irregular cations with small anions. Hence, aprotic ILs are good candidates for lithium batteries applications due to their high mobility and ion concentration.

Protic ILs result from combining a Brønsted acid and a base. The hydrogen-bonded network is formed by the availability of proton acceptor and donor sites caused by the migration of the protons from the acid to the base. Protic ILs could be formed with protons existing on the cation, such as triethylammonium bromide, 1-methylimidazolium chloride, and pyridinium bromide [19]. Another method for forming protic ILs is by attaching acidic functional groups such as sulfonate or sulfone-amide to the cation as the basis of protons in the architecture. Another type of protic ILs called multiprotic ILs are characterized by the presence of a functional group and a proton on the anion or the cation. These ILs are referred to as multiprotic since multiple protons exist in their structure.

### 3.1. Preparation of Polymerized Ionic Liquid Membranes

**Figure 2:**
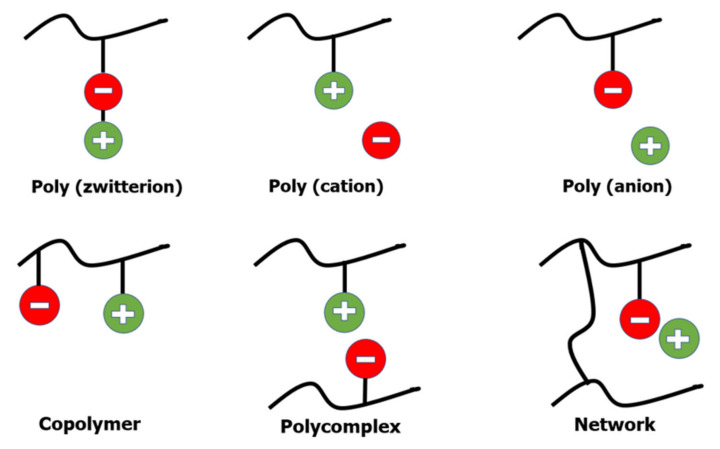
Depiction of poly (ILs).

Polymerization of ILs containing a polymeric group appears to be the most commonly used technique for creating polymeric electrolytes. Polymerised ILs (PILs) comprise a wide variety of structures depending on the nature of the final application. Figure 2 shows a summary of poly(ILs).

Mainly, PILs can be synthesized by direct polymerization of IL monomers or by chemical modification of existing polymers. Direct polymerization can be achieved by techniques such as free or controlled radical polymerization, reversible addition–fragmentation chain transfer polymerization (RAFT), and atom transfer radical polymerization (ATRP). The IL monomers that are usually employed in this method include methacryloyl, styrenic, and 1-vinylimidazolium groups. Controlled radical polymerization offers the advantage of designing and controlling the macromolecular construction of the IL species [18]. The ATRP and RAFT, on the other hand, are mainly utilized for the preparation of homopolymers and block copolymers of IL. Besides these, photopolymerization can be used, which is an intuitive method for the formation of PILs composites based on imidazolium-based monomer for application of gas separation membranes [35]. The PILs synthesized using the chemical alteration of existing polymers will adopt the structure of the original polymer in addition to a certain degree of polymerization.

The ionic conductivity of the IL monomer decreases as the glass temperature increases and the mobility and number of mobile ions decreases. The decrease in ionic conductivity is the most common disadvantage of PILs. This limitation can be addressed by increasing the concentration of carrier ions or modifying the polymer structure to increase the mobility of ions. For example, Põhako-Esko et al. [36] investigated the addition of 1-ethyl-3-methylimidazolium tetrafluoroborate [EMIM][BF_4_] to methacrylate-type PILs. It was reported that an increase in the amount of [EMIM][BF_4_] resulted in an increased ionic conductivity (0.1 mS/cm compared to only 0.01 mS/cm for the unmodified PIL).

**Figure 3:**
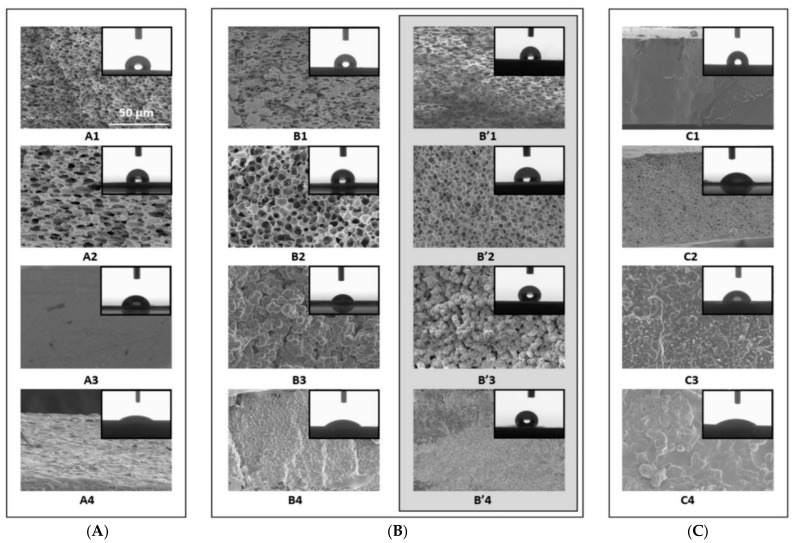
Cross-sectional SEM images for of PVDF-HFP membranes (1), PVDF-HFP/[MIm][Cl] (2), PVDF-HFP/[dema][TfO] (3) and PVDF-HFP/[MIm][NTf_2_] (4), prepared at room temperature (**A**), 80 °C (**B**) and 100 °C (**C**). B′ represents the membranes prepared at 80 °C after immersion in water for 48 h [37], Reproduced with permission from Paula GSaiz, et al., Materials & Design; published by Elsevier, 2018.

The addition of the ILs manipulates the morphology of the membranes, where ILs are known to be structure-directing agents. For instance, GSaiz et al. [37] studied the effect of ILs on the morphology of a PVDF-HFP membrane. The Scanning Electron Microscopy (SEM) results showed the presence of several morphologies and different hydrophobic properties depending on the selected IL. Figure 3 shows the cross-sectional SEM view of PVDF-HFP-based membranes with the presence of ILs. It can be seen that the pristine PVDF-HFP membranes show a high degree of porosity; however, membranes with ILs possess a different structure. For instance, PVDF-HFP/[dema][TfO] composites show no pores when prepared at room temperature, or heated at 100 °C. Nevertheless, a spherulites structure is observed when the solvent evaporates at 80 °C. Meanwhile, PVDF-HFP/[MIm][NTf_2_] composites showed no porosity. This indicates that hydrophilic ILs induce porosity when compared to hydrophobic ones. In general, the addition of ILs improves the morphology of the membranes as they reduce the occurrence and extent of variations on the membrane that causes local heat spots and leads to holes in membranes [38,39].

### 3.2. Ionic Liquids as Electrolytes in PEMFCs

Protic ILs represent promising candidates in PEM membranes due to several advantages including thermal stability, low volatility, and high conductivity above 100 °C. As established, Protic ILs are in base–acid equilibrium since they are created by the neutralization reaction between an acid and a base. Hence, the protic IL’s structure consists of a neutral species, a cation, and an anion. The neutral species are formed by the transport process of the back proton, where in case of incomplete proton transport, the ILs’ boiling could occur prior to the decomposition resulting in a decrease in the thermal stability, especially at high temperatures.

The proton transfer occurs via Grotthuss and vehicle mechanisms [19]. In the former mechanism, the proton moves from one water molecule to another utilizing the availability of hydrogen bonds. In the vehicle mechanism, the protons move by migration. In some cases, both mechanisms can coincide in one system. For protic ILs, the proton transport is generally conducted by the vehicle mechanism, as there are no sites in the structure to receive the protons, therefore the cations carrying active protons travel to transport those protons. In protic ILs containing hydrogen phosphite and hydrogen sulphate anion, the anions can serve as a proton donor or acceptor, hence allowing the coexistence of the Grotthuss and vehicle mechanisms. Nonetheless, the viscosity affects the ionic conductivity of the protic ILs [40].

The employed IL greatly affects the performance of PEMFC. The effect of the IL on the PEM can be easily studied by observing the values of the open circuit potential (OCP). The OCP mainly depends on the solubility and mass transfer coefficient of the reactants oxygen (O_2_) and hydrogen (H_2_), where a higher OCP means better performance. Miran et al. [41] were among the researchers who studied hydrogen fuel cells and acid dissociation constant (pKa) and its influence on proton transfer from acids to bases in protic ILs. They concluded that the OCP increased with increasing equilibrium constant up to 17, after which it decreased. This led to the conclusion that the proton migration is hindered by the value of pKa and the strong N-H bond.

Numerous research has been conducted to study the feasibility of employing protic ILs as proton conductors for PEMFC. Table 2 shows the conductivity values of common protic ILs used in fuel-cell applications. Nakamoto et al. [42] prepared protic ILs and melts by combining Benzimidazolium (bzlm) and bis(trifluoromethanesulphonyl)imide (TFSI) at different molar ratios. The resulting protic IL showed thermal stability at 350 °C and remained stable under oxygen reduction and hydrogen oxidation reactions. The measured conductivity at 140 °C was 8.3 mS/cm. Moreover, it was shown that [Bzlm][TFSI] melts can be utilized as an electrolyte at 150 °C for dry conditions. Nevertheless, at identical operating parameters, H_3_PO_4_ electrolyte exhibited larger open-circuit potentials than [Bzlm][TFSI]. Noda et al. [43] prepared protic IL using imidazole (Im) and TFSI at various molar ratios. The resulting protic IL showed thermal stability at 300 °C and above while increasing the oxygen reduction and hydrogen oxidization reactions. Nonetheless, the adsorption of imidazolium on the electrode caused the electric current to decline. Wippermann et al. [44] produced an IL of (2-sulfoethylammonium trifluoromethanesulfonate) [2-sea][TfO] (95 wt%) dissolved in water that displayed a conductivity 20 times less than H_3_PO_4_ at a temperature of 130 °C. However, at a lower temperature of 90 °C, the solution showed a higher activity for oxygen reduction reaction when compared to H_3_PO_4_. Moreover, protic IL [2-FPy]TF improved the fuel performance by displaying an increase of 15% in the polarization curve represented for a 0.1 V cell potential increase at 80 °C [45].

Some attempts have been made to improve the physical and chemical properties of protic ILs. This includes the incorporation of organic and inorganic compounds. For instance, [EMIM]HSO_4_ was tested as an electrolyte in a PEMFC [59]. The results showed that [EMIM]HSO_4_ is a promising electrolyte especially at elevated temperatures above 145 °C. [EMIM][TFSI] has also been tested as an electrolyte for PEMFC, and it showed good thermal stability for temperatures of 350 °C and above with a conductivity of 5.4 mS/cm [63]. 1-butyl-3-methylimidazolium tetrafluoroborate [BMIM][BF_4_] has been tested as an electrolyte for fuel-cell applications and displayed a conductivity of 1.2 mS/cm [64]. Li et al. [65] established hybrid PEMs based on [dema][TfO] and SiO_2_ monoliths. The membranes showed extremely high conductivity of 10 mS/cm; however, the membrane brittleness required improvement. Moreover, clay multistructures were studied due to their low cost, ease of availability, and high sorption capacity. Takashi et al. [66] intercalated three different ILs into montmorillonite clay, namely 1-ethyl-3-methylimidazolium octylsulfate [EMIM][OCSO_4_], N,N,N-trimethyl-N-propylammonium bis (trifluoromethanesulfonyl) imide [TMPA][TFSI], and N,N-diethyl-N-methyl-N-(2-methoxyethyl) ammonium bis (trifluoromethanesulfonyl)imide [DEMET][TFSI]. The ionic conductivities achieved for [DEMET][TFSI] and [TMPA][TFSI] were 1.88 and 1.99 mS/cm, respectively. Protic ILs based on morpholinium (N-ethylmorpholinium, N-methylmorpholinium, and morpholinium cations) were synthesized by Yoshizawa-Fujita et al. [49]. In their work, the protic ILs showed proton conductivities around 10–16.8, 21–29, and 1 mS/cm at 25, 100, and 150 °C, respectively.

The previous literature shows that ILs are promising alternatives to conventional electrolytes as proton conductors for PEMs. The main attractive feature of utilizing ILs as electrolytes is the high conductivities they possess at high temperatures, even in anhydrous conditions. The ILs are synthesized as film-like membranes by supporting them on polymers for PEMFC applications. In the following sections, the various polymers used in PEMFCs are discussed.

### 3.3. Polymer-Ionic Liquids Membranes

Solid-state electrolytes are the desirable method for preparing ILs electrolytes since the film structure is required by electronic devices. In order to achieve the film-like membrane, IL can be mixed with ordinary polymers. This results in a change in the glass transition temperature and enhances the properties of hydrogen transport. Nevertheless, this technique comprises the properties of the IL and the strength of the membrane [67]. An example of the technique is the polymerization of methylmethacrylatein1-ethyl-3-methylimidazolium bis(trifluoromethanesulfonyl)imide ([C_2_mim][TFSI]) in the presence of a cross-linking agent to synthesize a self-standing polymer gel with a proton conductivity of 10 mS/cm at room temperature [68]. In the following sections, we provide a review of all types of membranes that have been investigated with ILs. It should be noted here that these membranes can be classified into sulfonated fluorine-based (e.g., Nafion) and hydrocarbon-based membranes. Hydrocarbon-based membranes are generally categorized into aliphatic-based (such as polyimide) or aromatic-based, such as PBI. They are generally characterized by low proton conduction properties as opposed to the PFSA membranes. In addition, there is a difficulty encountered in forming a distinct phase between the hydrophilic and hydrophobic regions, which is another drawback that has limited their application in PEM fuel-cell membranes. Therefore, the most widely used hydrocarbon-based polymer materials that also involve ionic liquids include Nafion, PBI, PI, PVDF, PMMA, and PSS.

#### 3.3.1. Nafion-Based PEM

The conductivity of Nafion membranes mainly depends on their water content. A dramatic decrease in Nafion’s conductivity is observed for temperatures higher than the boiling point of water. Since high temperature is preferred in PEMFCs due to several advantages, including enhanced kinetics and better water management, Nafion membranes have been modified with a variety of materials including ILs. Diaz et al. [69] studied the behaviour of Nafion 112 membranes modified with 1-butyl-3-(4-sulphobutyl)-imidazoliumtri-fluoromethanesulphonate ([HSO_3_-BBIm][TfO]) and 1-methyl-3-(4-sulphobutyl)-imidazolium bis(trifluoromethylsulphonyl)imide([HSO_3_-BMIm][TFSI]) ILs. The presence of the electrochemically stable imidazolium cation and the highly conductive anions allowed for the enhanced performance of these membranes. The existence of ([HSO_3_–BBIm][TfO]) improved the current density of the fuel-cell membrane to 217 mA/cm with the humidification of the inlet gasses. Yuso et al. [70] explored the properties of Nafion 112 membranes combined with n-dodecyltrimethylammonium (DTA^+^). The optimum volume fraction occurred at 68% DTA^+^ after 22 h of contact and resulted in higher thermal stability when the membranes are compared at 120 °C. Noto et al. [71] studied Nafion 117 membranes modified with triethylammonium methanesulphonate (TMS) and triethylammonium perfluorobutanesulphonate (TPFBu). The addition of the IL increased the uptake of the membranes (20 and 39 wt.% for TMS and TPFBu, respectively). The synthesized membrane showed thermal stability up to 140 °C. Sun et al. [72] evaluated the conductivity of anhydrous Nafion membranes impregnated with imidazole-based molten salts in addition to three ILs, namely: 1,2-dimethyl imidazolium trifluoroacetate, imidazolium tri-fluoroacetate, and 1, 2-di-methyl imidazolium trifluoromethane sulfonate. The conductivity was high, at around 3–4 mS/cm. Sood et al. [73] modified Nafion membranes with triethylammmonium trifluorosulfonate. It was found that an increase in the triethylammmonium trifluorosulfonate (TEA-TF) content led to enhanced membrane conductivity and water uptake properties.

The most important factor to consider while manufacturing polymerized IL electrolytes is the compatibly between the IL and the polymer, as it greatly affects the uptake degree of the IL by the polymer. For instance, Schauer et al. [74] examined the compatibility of protic IL, 1-ethylimidazolium trifluoromethanesulfonate [EIM][TfO] and aprotic IL, 1-butyl-3-methylimidazolium trifluoromethanesulfonate [BMIM][TfO] with Nafion, and other polymers including Udel-type polysulfone, polybenzimidazole derivative containing benzofuranone moieties (PBI-O-Ph), and poly(vinylidene fluoride-co-hexafluoropropene) (fluoroelastomer). The results showed that owing to the low solubility of the two IL, they were completely incompatible with the polysulfone polymer. Thus, the conductivity was negligible, as the ionic conductivity structure did not form due to the low solubility. However, in the case of PBI-O-Ph, the IL was partially compatible. Hence, low conductivities were recorded for temperatures above 90 °C. In the case of charged polymers such as Nafion, a marginally higher conductivity was recorded. Lojoiu et al. [75] evaluated the effect of different ILs on Nafion conductivity and found a direct proportional relationship between conductivity and IL uptake. Hence, to maximize the conductivity of the membrane, optimization of the uptake of the IL by the polymer is an important consideration. For instance, [HSO_3_-BBIM][TfO] showed an uptake degree of 9.4% by Nafion, which is higher when compared to 1.6% for [HSO_3_-BBIM][TFSI]. Consequently, the performance of the resulting membranes was enhanced and a current density of 0.217 A/cm was observed [69]. Zanchet et al. [76] fabricated Nafion composite membranes and varied the ILs content, namely 3-triethylammonium propane sulfonic acid tetrafluoroborate (TEA-PSBF_4_), 3-triethylammonium propane sulfonic acid hydrogen sulphate (TEA-PSHSO_4_), and 3-triethylammonium propane sulfonic acid trifluoromethanesulfonate (TEA-PSCF_3_SO_3_). The ILs were reported to improve the proton conductivity and stability of the resulting membrane. The results showed that the conductivity increased from 0.093 S/cm (for unmodified Nafion) to 0.159 S/cm for 5 wt.% (TEA-PSHSO_4_)/Nafion at 80 °C.

The proton transfer mechanism between the anion and the cation in polymerized IL membranes was also investigated in the literature. Kumar and Venkatnathan [77] investigated the proton transfer in polymerized IL membranes in an attempt to aid the selection process of a suitable IL that will boost the conductivity of the polymer. It was reported that the modification enhanced the proton conduction paths. The conductivity of the Nafion/IL membranes was also affected by the dispersion of the IL within the membrane matrix. Under anhydrous conditions and at a temperature of 130 °C, the conductivity was 6 mS/cm [78].

**Figure 4:**
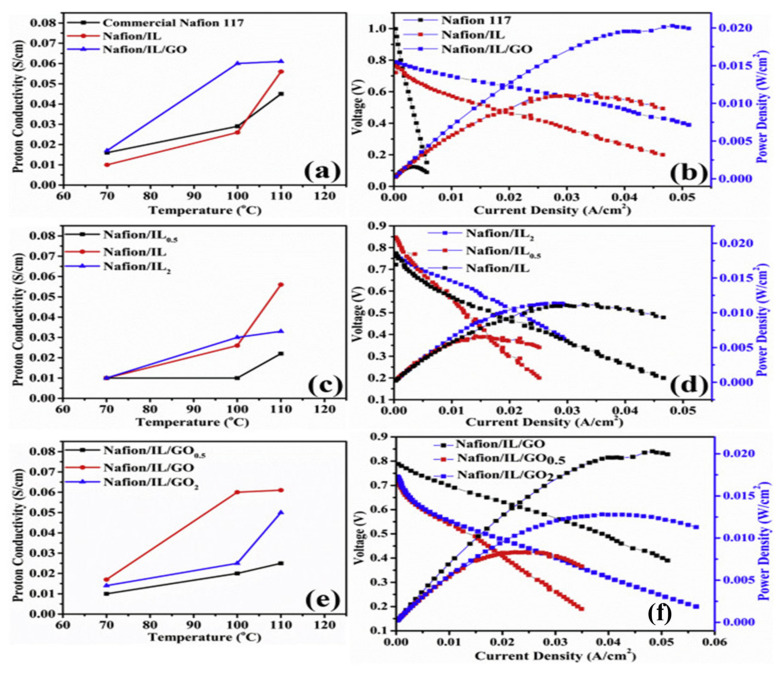
(**a**) Proton conductivities at different temperatures under anhydrous conditions. (**b**) I–V and power density, fuel: H_2_, operating conditions: 110 °C, 1 atm. (**c**) Proton conductivities of Nafion/IL membranes at different temperatures under anhydrous conditions, (**d**) I–V and power density curves of Nafion/IL membranes, fuel: H_2_, operating conditions: 110 °C, 1 atm. (**e**) Proton conductivity of Nafion/IL/GO membranes at different temperatures under anhydrous conditions and (**f**) I–V and power density curves of Nafion/IL/GO membranes, fuel: H_2_, operating conditions: 110 °C, 1 atm [82], Reproduced with permission from Elsevier, 2017.

The addition of silica (SiO_2_) showed an improvement in membranes along with ILs. For example, for Nafion/(SiO_2_)_3.67_(TEA)/(TEA-TF)_1.2_, a conductivity of 4.7 mS/cm was reported at 105 °C [79]. Another example is the Nafion/1-(3-hydroxypropyl)-3-methylimidazolium tetrafluoroborid [C_3_OHMIM]BF_4_/SiO_2_ (50/10 wt%) membrane, which was found to be stable up to a temperature of 405 °C due to the formation of hydrogen bonds. Moreover, the membrane achieved a conductivity of 55 mS/cm above 160 °C and a 100% increase in the tensile strength compared to pristine Nafion [80]. In another study, the protic IL1-(3-trimethoxysilylpropyl)-2-methylimidazolium tetrafluoroborate [TMSPMIM]BF_4_ was employed to functionalize mesoporous SiO_2_ in order to prevent IL retention. The resulting Nafion membrane showed a 7-fold increase in conductivity when 3 wt% functionalized SiO_2_ were utilized, where a conductivity of 375 mS/cm was obtained [81]. The use of graphene oxide (GO) to enhance the conductivity of Nafion-based membranes, especially at higher temperatures, has been investigated. Maiti et al. [82] synthesized a membrane using GO, [DMBIM]H_2_PO_4_, and Nafion 117. At 110 °C, the conductivity was 6.1 mS/cm. Figure 4 shows the influence of temperature on the proton conductivity of the membranes, as well as the effect of the current density on the voltage and power density of the PEMFC operating with hydrogen at 110 °C. The figure shows an enhancement in conductivity and fuel-cell performance (as seen by the polarization curve) upon the modification of Nafion using ILs or additives (e.g., GO).

The structure and length of the side chain of fluorine-based membranes significantly affect the conductivity of the polymer membrane. Fluorine-based polymer electrolyte membranes can also include other types such as Aciplex and Flermon membranes, but to the best of our knowledge, they have not yet been used in PEM fuel-cell membranes with ILs in particular. When it comes to the side chain, and upon the examination of fluorine-based membranes, it can be clearly seen that Nafion is characterized by a long side chain. Aquivion (Solvay) and PFSA (3M) ionomers, on the other hand, have short side chains. The short side chains appear to exhibit more durability, however, they can also affect the crystallinity and eventually the glass transition temperature. PFSA (3M) ionomers were found to be investigated in only one study that involved the modification with imidazole ionic liquid [83]. The limited number of studies is probably due to their poor mechanical properties and decreased conductivities as opposed to Nafion.

In general, the strategy to improve the conductivity while maintaining the mechanical properties of the semicrystalline backbone is usually by the utilization of a multi-acid side chain. For instance, some side chains contain two sulfonyl fluoride groups that are hydrolysed to sulfonic acid. Others rely on bis(perfluooalkylsulfonyl)imide which competes with the acid strength of a perfluoroalkyl sulfonic acid. A recent development is the production of perfluoroimide acid (PFIA) ionomers with the addition of several bis(sulfonyl)imides added to the side chain to produce perfluoroalkyl ionene chain extended (PFICE) ionomers [84].

The previous literature has summarized the recent work on Nafion/IL membranes. Researchers mainly discussed the enhancement in the properties of the Nafion membranes caused by incorporating IL. In this context, the compatibility of IL and the polymer matrix is an important consideration that affects the degree of uptake of the IL by the polymer, and consequently, the conductivity of the resulting membrane. Table 3 shows a summary of the conductivity of Nafion-based membranes.

#### 3.3.2. Polyvinylidene Fluoride (PVDF)-Based PEM

Polyvinylidene fluoride (PVDF) and its copolymers are widely used in polymer/IL blends. These polymers are characterized by the production of stable film possessing outstanding thermal and mechanical properties, since the presence of an amorphous region allows for a large number of liquid electrolytes. Martinella et al. [85] studied the possibility of using PVDF-HFP as a matrix polymer for EIMTFSI by investigating the thermophysical properties of polymer/IL membrane. It was demonstrated that by increasing the polymer concentration, the thermomechanical stability increases. The conductivity was measured to be 10 mS/cm for the highest IL content of 80 wt.%. Lee et al. [86] synthesized composite membranes using 1-ethyl-3-methylimidazolium fluor hydrogenates [EMIM](FH)_n_F) ILs and the fluorinated polymer compounds (S-DFBP-HFDP) and (PVDF-HFP). The conductivity of the membrane that had a weight ratio of 1/0.3/1.75 was around 34.7 mS/cm at 130 °C. Furthermore, the reported open-circuit voltage, power density, and current density were reported to be 1 V, 0.020 W/cm^2^, and 0.06 A/cm^2^, respectively. Gao et al. [87] examined the performance of a PVDF/IL composite membrane by varying the following cations: [Epdy], [N1114]^+^, and [EMIM]. Using [N1114] HSO_4_, a 60-fold increase in maximum power density was observed when compared to imidazolium-based IL. The incorporation of the protic IL and PVDF-HFP resulted in a composite membrane with a high melting temperature and a conductivity of 10 mS/cm at 140 °C [52]. Superacids have also been studied to complement the composite membranes, as they are famous proton conductors and carriers. Nair and Mohapatra [88] synthesized HClO_4_SiO_2_/[dema][TfO]/PVDF-HFP composite membranes. The SiO_2_ particles were employed to foil the leak of [dema][TfO] from the membrane, in addition to serving as a substrate to immobilize HClO_4_ that was utilized for conductivity enhancement. The optimum weight percent of [dema][TfO] occurred at 80 wt.%, where the recorded conductivities of the membrane were 0.02 and 0.6 mS/cm at 30 and 100 °C, respectively. Protic IL based on amides has also been utilized. For instance, at 150 °C, ITSA exhibits a high ionic conductivity of 32.6 mS/cm. Xiang et al. [53] studied the physicochemical properties of ITSA/PVDF. A higher amount of PVDF decreased the conductivity. This issue was solved by the addition of PAI as a polymeric additive. Thus, an ionic conductivity of 7.5 mS/cm was achieved for PAI/PVDF (60/5 wt.%) composite membrane.

In case of PVDF-HFP membranes, the conductivity can be enhanced by incorporating additives such as aluminium oxide particles and Polyethylene glycol (PEG) [89]. Another method is the addition of copolymers to the PVDF/IL composite membrane. Inan et al. [90] combined SPEEK with PVDF-HPF membrane and observed that the conductivity and mechanical and chemical stabilities were enhanced. Nanocomposites such as titania and silica have also been incorporated with PVDF-HPF membrane to produce similar enhancements [91,92]. Mališ et al. [93] synthesized polymer/IL membranes by mixing PVDF-HPF and Nafion polymers with BMIMTfO and EIMTfO aprotic and protic IL, respectively. The synthesized membranes were employed in fuel cells (90–160 °C) and compared with PBI reference fuel cells. The conductivity of the composite membranes, especially those based on EIMTfO, increased with the presence of water. For instance, adding 5 wt.% water increased the ionic conductivity by 170% to a value of 13.5 mS/cm. The operating conditions played a vital part in the performance of the composite membranes. Under wet conditions, Nafion/BMIMTfO membranes displayed the most superior conductivity, mainly due to the weak interaction between BMIMTfO and the polymer. However, in dry conditions, the highest conductivity observed was for PVDF-HFP/EIMTfO membrane due to the late interaction between the polymer and IL, which permitted the water to stay in the structure, hence causing the superior conductivity. Nevertheless, the fuel cell did not outperform the PBI reference cell. Table 4 shows a summary of the conductivity of PVDF-based membranes.

#### 3.3.3. Polybenzimidazole (PBI)-Based PEM

PBI is commercially available and is the furthermost widely investigated and utilized polymer for high-temperature fuel-cell applications [94]. This is attributed to its low cost and good thermal and mechanical stabilities. Eguizábal et al. [95] synthesized hybrid membranes containing 1-H-3-methylimidazolium bis(trifluoromethanesulfonyl)imide [HMI][TFSI] and PBI polymer for high-temperature PEMFCs application. The optimal membrane composition achieved a conductivity of 54 mS/cm at 200 °C. Van de Ven et al. [96] combined 1-H-3-methylimidazolium bis (trifluoromethanesulphonyl)imide with PBI to produce a hybrid membrane with a proton conductivity of 1.86 mS/cm at 190 °C, exceeding that of Nafion 117. Ye et al. [97] produced a composite membrane based on H_3_PO_4_/1-propyl-3-methylimidazolium dihydrogen phosphate (PMIH_2_PO_4_)/PBI for high-temperature PEMFCs. The PEMFC exhibited a proton conductivity of 2.0 mS/cm when operated at 150 °C under anhydrous conditions. Liu et al. [98] investigated the compatibility between PBI and protic IL diethylmethylammonium trifluoromethanesulfonate [dema][TfO], and developed a hybrid membrane exhibiting a conductivity of 20.73 mS/cm at 160 °C. Wang et al. [99] created a cross-linked structure PBI-IPTS grafted to PBIOH30 backbone (IPTS30)/1-butyl-3-methylimidazolium dihydrogenphosphate [BMIM]H_2_PO_4_ hybrid membranes. Due to the aromatic ring structure of the [BMIM]H_2_PO_4_ cation, the compatibility with PBI was increased, which consequently enhanced the proton conductivity. The measured conductivity at 160 °C was 133 mS/cm. Nevertheless, the presence of the cation increased the spacing between the polymeric chains leading to a reduction in the mechanical strength. For instance, compared to neat PBI membranes, a 35% decline in tensile strength and a 30% decline in Young’s modulus were observed for PBI/[BMIm]H_2_PO_4_ (10 wt.%).

Protic ILs have also been added to porous PBI to form composite PEMs. Eguizábal et al. [100] manufactured porous PBI treated with microporous titanosilicate (ETS-10) and combined with methylimidazolium bis(trifluoromethanesulfonyl)imide [MIM][TFSI] IL to form a PEM. The membrane achieved an in-plane conductivity of 100 mS/cm and a through-plane conductivity of 40 mS/cm at 160 °C. However, durability tests up to 500 h revealed a severe degradation in the proton conductivity, where values of 15 and 6 mS/cm were achieved at 150 and 200 °C, respectively. In addition, the rise in the temperature resulted in the improvement of the power and current densities of the composite membranes. For example, the rise in temperature from 25 to 180 °C raised the power and current densities by 96% and 200%, respectively, for compact PBI membranes at 0.5 V. Lemus et al. [101] prepared membranes utilizing porous PBI and 1-H-3-vinylimidazolium bis(trifluoromethanesulfonyl) imide [IMVH][TFSI]. Synthesis was performed using ultraviolet photopolymerization and reported conductivities were 371 mS/cm when synthesized with 2.5 mol% divinylbenzene (DVB) crosslinker and 309 mS/cm when synthesized with a pre-existing PBI with 80% porosity. The stability of conductivity tests showed a decrease in conductivity for the 500 h, and then the conductivity approximately stayed constant at a value of 250 mS/cm for both membranes. Kallem et al. [102] prepared PBI membranes infiltered with [IMVH][TFSI]. The presence of the IL repressed the transport of the proton, yet it improved both the thermal and mechanical stability. The measured conductivity at 200 °C was around 53.3 mS/cm for a hybrid membrane PBI/IL/DVB (0.88/58.5/1%). The tensile strength was reported to be 10.0 MPa. To enhance the membrane conductivity, [IMVH][TFSI] was added to hierarchical porous polybenzimidazole (HPBI). A conductivity of 85 mS/cm at 200 °C was obtained for a HPBI (49.5 wt.%)/IL (58.5 wt.%)/DVB (1%) composite membrane [103]. Nawn et al. [104] synthesized membranes consisting of polybenzimidazole membranes (PBI4N) impregnated with zirconium dioxide (ZrO_2_) nanofillers. The content of the ZrO_2_ filler varied between 0 and 22 wt.%. The results show that the content of the filler plays a vital role in the performance of the PEM. For instance, as the content increased from 0 to 8%, the thermal and mechanical stabilities increased. However, when increasing from 8 to 22%, the opposite effect is demonstrated, where the stabilities decreased. The [PBI4N(ZrO_2_)_0.231_] (H_3_PO_4_)_13_ membrane attained a conductivity of 104 mS/cm at 180 °C. Hybrid membranes consisting of PBI/[dema][TfO] were employed in fuel cells. The membrane showed a conductivity of 20.73 mS/cm at 160 °C. In addition, for PBI/[dema][TfO], membranes showed a conductivity reduction of 10% (with 83 wt.% [dema][TfO]) and 18% (with 80 wt.% [dema][TfO]) after 400 hr. Moreover, PBI/[dema][TfO] (83 wt.%) membrane exhibited power density of 26.50 and 29.64 mW/cm^2^ at 120 °C and 140 °C, respectively [98]. Xu et al. [105] prepared PEM by incorporating IL graphite oxide (ILGO) with PBI loaded with polymer acid (PA). At 175 °C, the proton conductivity was measured as 35 mS/cm, while the power density was measured to be 320 mW/cm^2^. It was concluded that ILGO filler could enhance the conductivity of the PEM. Hooshyari et al. [106] developed composite membranes incorporating PBI with 1,3-di(3-methylimidazolium) propane bis (trifluoromethylsulfonyl) imide (PDC_3_) and 1-hexyl-3-methylimidazolium bis (trifluoromethanesulfonyl) imide (PMC_6_) dicationic IL. The membrane was tested for PEMFC applications under anhydrous conditions and 180 °C, and PBI/PA/PDC_3_ attained an ionic conductivity and power density of 78 mS/cm and 400 mW/cm^2^, respectively. Liu et al. [107] synthesized functional silica IL into PBIOH to produce composite membranes for PEMFC applications. Subsequent to investigating the trade-off between the conductivity and stability of the PEM based on the weight percent of the IL, an optimum content of 5 wt.% was reached. At that ratio, the proton conductivity was measured as 106 mS/cm at 170 °C.

For PBI/IL composite membranes, numerous studies showed that proton conductivity improved and remained stable for a longer duration. However, the presence of IL amplified the space between the polymeric chains of PBI and caused a deterioration in the intermolecular forces and mechanical strength. Table 5 shows a summary of the conductivity of PBI-based membranes.

#### 3.3.4. Sulfonated Poly (Ether Ether Ketones) (SPEEK)-Based PEM

SPEEK membranes are widely investigated in PEMFC owing to their high thermal and chemical stability and their ability to retain water to enhance proton conductivity. Jothi and Dharmalingam [108] prepared and tested SPEEK/1-ethyl-3-methylimidazolium diethyl phosphate [EMIM][DEP] membrane by casting. Under anhydrous conditions at 145 °C, an ionic conductivity of 3 mS/cm was recorded as well as a power density of 203 mW/cm^2^ with OCP of 0.83 V. Li et al. [109] added silicon oxide particles SiO_x_ to a SPEEK/[dema][TfO] membrane and investigated the resulting hybrid membranes. The addition of the SiO_x_ decreased the IL leakage and the inflammation degree of the membrane due to the promoted interaction between SPEEK, the IL, and siloxane. Under dry conditions, the SPEEK based membrane showed a conductivity of 20.03 mS/cm at 220 °C. Sulfonated SPEEK/[dema][TfO] IL/with modified montmorillonite clay and dema+ cation (Mmtdema) exhibited an ionic conductivity of 78 mS/cm at 70 °C [110]. Wang et al. [111] introduced mesoporous SiO_2_ to SPEEK/IL membranes and discovered that the presence of silica improved the IL preservation in SPEEK. For instance, SPEEK/[BMIM][BF_4_]/SiO_2_ (50 wt.%/7.5 wt.%) membranes displayed a conductivity of 15 mS/cm. Batalha et al. [112] improved the conductivity of SPEEK/[dema][TfO] composite membranes by the addition of ZrO_2_. The interaction between the IL and the elongated architecture of zirconia allowed for the existence of new proton transport channels. For instance, the proton conductivity of SPEEK increased from 70 to 660 mS/cm with the addition of [dema][TfO] and 6 wt% of ZrO_2_. SPEEK and [BMIm]^+^ cations show excellent interaction, which allows for the retention of IL by SPEEK [113]. For example, at 160 °C and dry conditions, the hybrid membrane SPEEK/1-butyl-3-methylimidazolium hexafluorophosphate [BMIm]PF_6_ (50%)/PA (4.6 doping level) exhibited a conductivity of 30 mS/cm. The durability test displayed a stable conductivity of 20 mS/cm for periods more than 600 hr. da Trindade et al. [114] aimed at enhancing the SPEEK membranes’ performance by the addition of (ImHSO_4_), (MIHSO_4_) and BMIHSO_4_ IL. The conductivity increased by 50% and 30% when the membranes were doped with 5 wt.% of (MIHSO_4_) and BMIHSO_4_, respectively. Moreover, for PEMFC applications SPEEK/BMIHSO_4_ PEM showed a power density of 530 mW/cm^2^ in the range of 80 to 100 °C. Che et al. [39] prepared a multicomponent hybrid membrane based on SPEEK, polyurethane (PU), and BMIm^+^ IL using layer-by-layer fabrication. After 100 layers, the PEM was obtained and doped on PA to be tested for PEMFC. The PEM (SPEEK/PU/SPEEK/BMIM)_100_/60%PA exhibited conductivity and tensile strength of 103 mS/cm (at 160 °C) and 2.38 MPa, respectively. Elumalai et al. [115] synthesized composite membranes based on phosphonate IL immobilised mesoporous silica SBA-15 as a filler to the SPEEK polymer matrix. In this case, the observed conductivity was 10.2 mS/cm with 6% SBA-15/SPEEK at 140 °C. At the same temperature, the mechanical strength was measured as 23 MPa, while a power density of 183 mW/cm^2^ was also obtained. Table 6 shows the conductivity values of some SPEEK-based membranes.

#### 3.3.5. Sulfonated Polyimide (SPI)-Based PEM

Among other promising polymers for PEMFC is sulfonated polyimide (SPI), which exhibits high mechanical strength, high thermal stability, and low permeability. Chen et al. [116] manufactured hybrid membranes by combining 1-vinylimidazolium trifluoromethanesulfonate [ImVH][TfO], 1-methylimidazolium trifluoromethanesulfonate [MIm][TfO] IL with SPI. The resulting polymer structure is strengthened due to ionic bonds formed between the IL cations and SPI sulfonic acid groups at elevated temperatures, in turn improving the polymer’s preservation of the IL, increasing the long-term stability and conductivity. Under dry conditions, SPI/[ImVH][TfO](50 wt.%) membrane showed an ionic conductivity of 3–6 mS/cm at 120 °C. Another composite membrane consisting of SPI/[MIm]BF_4_/H_2_SO_4_ was tested for fuel-cell applications [117]. The ionic conductivity achieved was 55.9 mS/cm at 180 °C, however, the value deteriorated to 28.2 mS/cm after three months. Lee et al. [118] tested SPI/[dema][TfO] (67 wt.%) membranes for PEMFC after confirming the compatibility between the IL and the polymer. Under dry conditions, the measured conductivity was 10 mS/cm at 120 °C. For PEMFC performance, a current density of 240 mA/cm^2^ and a power density of 100 mW/cm^2^ were achieved at 80 °C under dry conditions. Chen et al. [119] synthesized the hybrid membrane SPI/[ImVH][TfO] (40 wt.%) by employing 1,4-bis(4-aminophenoxy-2-sulfonic acid) benzenesulfonic acid (pBABTS), 3,3′, 4,4′-diphenyl sulfone tetracarboxylic dianhydride (DSDA) as dianhydride, and 4,4′-(9-fluorenylidene) dianiline (9FDA) as diamine. Under dry conditions, the PEM attained a proton conductivity of 16 mS/cm at 120 °C. Kowsari et al. [120] synthesized PEM doped with PA and constructed on SPI/graphene oxide (GO). The GO was functionalized with [MIm]^+^ and H_2_PO_4_^−^. The measured conductivity of the membrane at 160 and 120 °C were 77.2 and 124.3 mS/cm, respectively.

#### 3.3.6. Other Polymer-Based PEM

**Figure 5:**
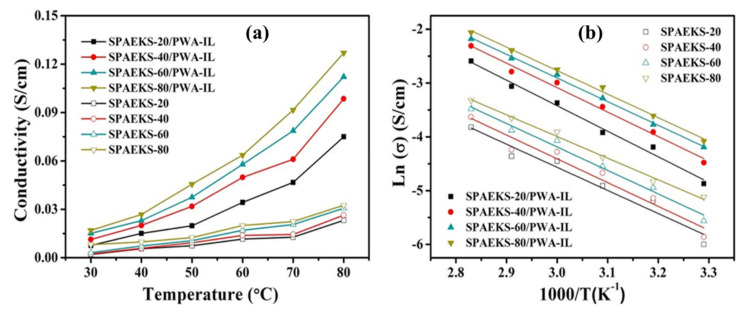
(**a**) Proton conductivity and (**b**) Arrhenius plot (right) of SPAEKS-X membranes and SPAEKS-X/PWA-IL membranes at different temperatures [122], Reproduced with permission from Jatindranath Maiti, et al., Composites Science and Technology; published by Elsevier, 2018.

Zhang et al. [121] synthesized a hybrid membrane by incorporating 1-butyl-3-methylimidazolium bis(trifluoromethanesulfonyl)imide [BMIm][TFSI] IL with silicon nanorods (SNR) into poly(2,5-benzimidazole) (ABPBI) polymer. The conductivity was 10 mS/cm under a temperature range of 80–140 °C. The PEMFC performance attained power densities of 150 and 280 mW/cm^2^ at 80 and 180 °C, respectively. Li et al. [122] synthesized composite membranes using sulfonated poly(arylene ether ketone sulfone) (SPAEKS) and 1-ethyl-3-methyl imidazolium phosphotungstate (PWA-IL). At 80 °C, the PEM showed an excellent proton conductivity of 127 mS/cm. Figure 5 shows the variation of the temperature on the conductivity of SPAEKS-based membranes, in addition to the Arrhenius plot. Awasthi et al. [123] synthesized a hybrid membrane based on poly(arylene ether) polymer while incorporating [BMIm][BF_4_] IL. The hydrophilic length was constant at 8 kDa, while the hydrophobic length was varied (5, 8, and 10 kDa) to produce the polymers (named as P1, P2, and P3 polymers, respectively). The increase in the hydrophobic length resulted in a decline in the proton conductivity of the membrane owing to lower water retention. Moreover, the IL improved the conductivity. For instance, the proton conductivity of P1 increased from 32 to 75 mS/cm when 50 wt.% of IL was added to the PEM. Following the trend, the proton conductivity of P2 rose from 25 to 61 mS/cm and the proton conductivity of P3 increased by 109% with the addition of 50 wt.% [BMIm][BF_4_].

Dahi et al. [124] synthesized hybrid membranes based on Matrimid^®^ polymer and 1-n-butylimidazolium bis(2-ethylhexyl)phosphate [BIm][BEHP], 1-n-butylimidazolium dibutylphosphate [BIm][DBP], and 1-n-methylimidazolium dibutylphosphate [MIm][DBP] hydrophilic IL. The hybrid membranes display great stability as most of the IL is reserved in the pores of Matrimid^®^. In addition, the viscosity of the IL deteriorated with increasing temperature, causing leakage of the IL and decreasing the conductivity. The PEM [BIm][DBP]/Matrimid^®^ attained the most superior proton conductivity of 20 mS/cm when compared to its counterpart at 115 °C. Langevin et al. [55] prepared composite PEM by immersing segments of MAT14PVP7 in TEA-TF IL. The proton conductivity of the PEM at 130 °C was measured as 20 mS/cm, which is higher when compared to the proton conductivity of TEA-TF/Nafion standing at 1.8 mS/cm. The results also revealed that, unlike Nafion, the mechanical resistance of MAT14PVP7-based PEM is barely affected by the increase in temperature.

Zakeri et al. [125] developed and fabricated composite membranes based on [ETFE-g-poly(4-VP)-SO_3_H]HSO_4_. At 95 °C, the composite membrane revealed a staggering conductivity enhancement of 62% of that of the Nafion 112 membrane. The membrane also displayed high thermal stability. Moreover, Zakeri et al. [126] also manufactured a similar composite membrane structure of Poly(1-vinyl imidazole) grafted poly(ethylene-co-tetrafluoroethylene) Trifluoromethanesulfonate [ETFE-g-P(1-Vim)_Pr_so_3H_ ]CF_3_SO_3_. At 95 °C, the PEM revealed a proton conductivity of 138 mS/cm, with 96% retention at 80 °C and for 50 hr under fully hydrated conditions. Tang et al. [127] synthesized composite membranes using adsorbed and doped [MIM][TfO] over polyacrylamide/polyethylene glycol interpenetrated (PAM/PEG IPN). With the variation in the concentration of the IL (22.84 wt.%, 50 wt.%), the conductivities attained at 150 °C were 10.37 and 17.02 mS/cm, respectively. Tang et al. [128] prepared [MIM][TfO] into poly(acrylic acid)–poly(ethylene glycol) superabsorbent membranes with proton conductivities reaching 40.4 mS/cm. The tests were performed under dry conditions and at a temperature of 200 °C. Chopade et al. [129] studied the structure of N-ethylimidazolium bis(trifluoromethylsulfonyl)imide [EIM][TFSI]/PEO composite membranes. The thermal and mechanical stabilities were improved due to the cross-linked structured polymer. Hence, even at a high temperature of 180 °C the PEM with 45 wt% of [EIM][TFSI] exhibited a conductivity of 14 mS/cm. Chu et al. [130] synthesized elastic and clear composite membranes utilizing PAMAM dendrimer-based macromolecular IL. The PEM displayed elevated thermal stability until temperatures of 350 °C, and a conductivity of 12 mS/cm at 160 °C and under dry conditions. PEM based on [MIm][TfO] and [APMIm][Br]-GO with a 1.0 wt.% achieved a conductivity of 14.8 mS/cm at 160 °C [131].

Thanganathan and Nogami [132] improved the properties of poly(vinyl alcohol) (PVA) membranes by incorporating 1–butyl–3–methylimidazolium Bis (trifluoromethanesulfonyl)imide (BMITFSI) and 1–ethyl–3–methylimidazolium tetrafluoroborate (EMI-BF_4_) ILs. Silica was incorporated as well to improve the stability and conductivity of the PEM. The hybrid membranes displayed conductivities of a maximum of 0.58 mS/cm at 60 °C. Yang et al. [133] synthesized composite membranes based on (PVA)-Citric Acid (CA)-IL for PEMFC applications. The tested results proved that PVA-CA-IL membranes show excellent performance under anhydrous conditions. Furthermore, PVA-CA-EAN (1:0.05:0.4 molar ratio) exhibited the most superior proton conductivity of 7.8 mS/cm at 140 °C. Wu et al. [134] studied the effect of poly(phenylene oxide) (PPO)/methylimidazole (MeIM) composite membranes on the performance of a PEMFC. At 30 °C, the PPO/MeIM membrane recorded a proton conductivity of 67.9 mS/cm in addition to a mechanical strength of 4.8 MPa at a molar ratio of 4:10 Moreover, without the additional humidification the PEMFC displayed a power density of 260 mW/cm^2^ at 160 °C.

**Figure 6:**
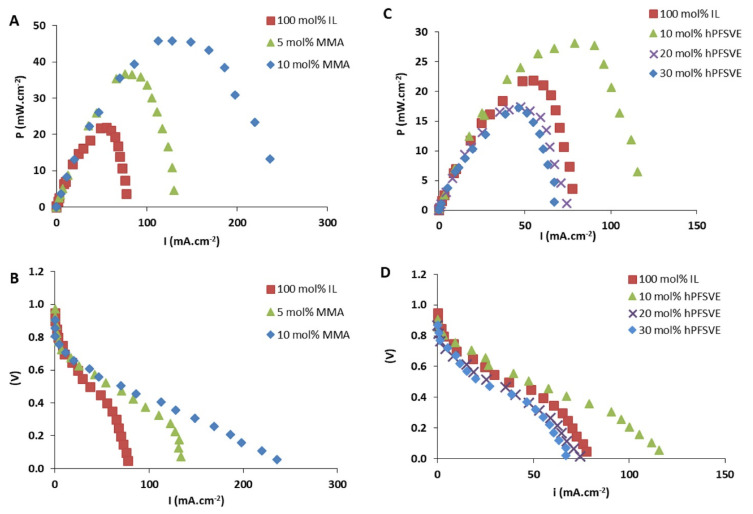
(**A**,**C**) Power curves and (**B**,**D**) polarization curves for PEMFC membranes based on poly(IL-co-hPFSVE), poly([IL-co-MMA), and poly(IL) [141]. Reproduced with permission from Elsevier, 2019.

The proton conductivity of Zirconium phosphate (ZrP) powder has been examined and composite membranes prepared from ZrP and porous polytetrafluoroethylene (PTFE) with and without mineral acids have been also investigated as electrolytes for direct hydrocarbon fuel cells that operate at high temperatures [135,136,137,138]. Moreover, Mohammed et al. [139] synthesized PEM based on ZrP and imidazolium-based IL for high-temperature fuel-cell applications. Five imidazolium based ILs were tested: 1-ethyl-3-methylimidazolium ethyl sulfate [EMIM][ESO_4_], 1-butyl-3-methylimidazolium dicyanamide [BMIM][DCA], 1-butyl-3-methylimidazolium triflate [BMIM][TFA], 1-ethyl-3-methylimidazolium acetate [EMIM][AC], 1-butyl-3-methylimidazolium Trifluoroacetic [BMIM][TFA]. ZrP/[EMIM][ESO_4_] recorded the most superior proton conductivity of 22.6 mS/cm when compared to its imidazolium-based IL counterparts. The reported results showed better performance than Nafion membranes, which highlights the great potential of ZrP-based PEM. A similar study by Al-Othman et al. [140] reported on the synthesis of composite membranes based on ZrP polymer, imidazolium-based IL, and glycerol supported on PTFE. The ZrP/[EMIM][SO_4_]/GLY/PTFE membrane was tested for PEMFC applications and demonstrated a proton conductivity of 70 mS/cm at 200 °C. Ortiz-Martínez et al. [141] copolymerized 1-(4-sulphobutyl)-3-vinylimidazolium trifluoromethanesulphonate [HSO_3_^−^BVIm][TfO] IL with methyl methacrylate MMA and perfluoro-3,6-dioxa-4-methyl-7-octene sulfonyl fluoride (hPFSVE) to form PILs for PEMFC applications. Both poly ([HSO_3_^−^BVIm] [TfO]-co-MMA) and poly([HSO_3_-BVIM][TfO]-co-hPFSVE) electrolyte membranes offered high conductivity in the range of (1–10 mS/cm) for wet and dry conditions. The performance of membranes was improved, showing the promising prospects of these PILs as PEM, with power outputs up to 45 mW/cm^2^. Figure 6 shows the performance of the PEMFC for various mall ratios.

Zhu et al. [142] prepared PILs with precursors of 1,3,5-tris(1-imidazolyl)benzene (TIB) and incorporated them into poly (2,6-dimethyl phenylene oxide) (QPPO) polymer matrix to produce hybrid PEM. The resulting TIB/QPPO hybrid membranes exhibited decent thermal stability, suitable mechanical properties, and improved conductivity and fuel-cell performance. For instance, the highest conductivity and fuel-cell performance recorded were 55 mS/cm and 168.9 mW/cm^2^ at 80 °C, respectively. Eisa et al. [143] prepared PEMFC based on polyaniline (PANI), 1-Hexyl-3- Methylimidazolium Tricyanomethanide IL, and ZrP. The proposed PEMFC with (50:50 mg) PANI/ZrP and 3.7 wt.% IL showed a promising ionic conductivity of 20 mS/cm at room temperature. Ka’ki et al. [40] synthesized and evaluated nanocomposite PEMFC based on calcium phosphate (CP) and ILs. PEMFC with CP/PTFE/[HMIM][C_4_N_3_^−^] composite membranes possessed a proton conductivity of 100 mS/cm at room temperature and 3.14 mS/cm at 200 °C. Table 7 lists the conductivity values of polymer-based membranes.

Sulfonated polyphenylene oxide (SPPO) has also been investigated as a polymer matrix for PEMFCs. Fu et al. [144] prepared several SPPO membranes for fuel-cell applications. The membranes showed a proton conductivity of 94 mS/cm at 25 °C, which is comparable to that of Nafion 117. Yang et al. [145] prepared SPPO-based membranes in N-methyl-2-pyrrolidone solution for PEM applications. The resulting membranes exhibited a proton conductivity of 11.6 mS/cm at 25 °C, which is also comparable to that of Nafion 112. Liu et al. [146] presented a method to stabilize poly(methyl-methylcrylate) (PMMA)-grafted magnetic nanoparticles with sulfonated polystyrene (SPS) in IL/cosolvent mixtures. The experimental results displayed that the highest ionic conductivity in the range of 30 mS/cm occurred while dissolving PMMA-PSS in acetonitrile and ILs. Zhang et al. [147] studied the enhancement of SPAEKS-based PEMS by the introduction of [EMIM][N(Tf)_2_] IL, MIL-100 metal organic framework, and phosphotungstic acid (HPW). Membranes based on SPAEKS with HPW-ILs@MIL-100 showed an outstanding conductivity of 138 mS/cm at 100 °C. Wang et al. [148] synthesized a series of sulfonated poly(arylene ether ketone ketone sulfone) SPAEKKS polymers for PEMFC applications. The resulting membranes reported a proton conductivity of 32 mS/cm at 80 °C, which is comparable to Nafion 117.

## 4. IL/Polymer PEM: Challenges and Future Work

The durability of PEM membranes is one of the most critical properties. It is affected by various factors that can cause membranes’ degradation either in mechanical, thermal, or chemical modes [149,150]. The mechanical degradation appears in the form of pinholes and cracks that are usually present as a result of manufacturing defects and can be detected in the early life of the PEMFC. The presence of cracks and pinholes lead to fuel crossover, hence, a combustion reaction resulting from the reaction of hydrogen and oxygen, instead of an electrochemical reaction. This leads to a severe safety risk [151]. The delamination of the membrane–electrode interface is another issue that has a direct influence on membrane durability of the PEMFCs. Delamination is a result of stress cycling and causes severe interfacial resistance, thus, an intense performance drop. The high-temperature operation is another serious issue that affects the durability of the PEM, as material decomposes around 280 °C for PFSA membranes [151]. Another issue caused by thermal degradation is the membrane thinning. The membrane’s material decomposition causes a reduction in the thickness of the membrane. Membrane thinning can lead to fuel crossover and electrical short circuits. The oxidization of the platinum catalyst under fuel-cell conditions is another important issue in fuel-cell materials’ durability [150].

In order for PEMFC technology to outperform the current technologies, the durability and performance ought to be increased, while decreasing the cost. The performance of the PEMFC highly depends on the power densities obtained. Therefore, an improvement in the conductivity of the PEM and a decline in the electrodes’ polarization losses will surely improve the power densities. Moreover, the PEM should meet the requirement of PEMFC application, especially the required stable performance for high-temperature PEM. Thus, the electrode/membrane interface (whether electrically or chemically) will affect the fuel-cell performance. For instance, the reduced interface will result in elevated electronic resistance and low utilization.

The main challenge facing polymerized IL membranes is the compatibility of ILs with the matrix polymers and the organic–inorganic fillers. There has been a clear compromise between the conductivity and the mechanical strength of the composite materials. Another major issue is the long-term thermal and mechanical stability of the composite membranes, especially under high-temperature PEMFC operation. The leakage of the IL from the membrane structure is another challenge faced in PEMFC operation. Moreover, PEM doped in acids to increase the conductivity should possess the ability to hold the acid, which in turn will improve the long-term stability of the PEM.

Research in the coming future must be directed towards studying several IL cations such as pyrrolidinium or triazolium, since all the present research fixated on ammonium and imidazolium-based IL has been exhausted. Poly ILs have the potential to provide a feasible alternative for conventional and commercially available polymers. Poly ILs are known for their excellent conductivity and high thermal stability, as well as their environmentally friendly and low-cost synthesis compared to Nafion [152]. Further, poly ILs are considered a promising replacement for IL as they are more stable and safe, possess better mechanical properties, and most importantly they offer a solution for the leakage issues. Hence, future research should be dedicated to investigating the employment of poly IL in PEMFC. The molecular and morphological structures of the PEM ought to be studied using molecular dynamics and orbital simulation, as they provide information that cannot be attained experimentally. Research should also focus on studying the proton conduction mechanisms inside the PEM to enable the design of IL-polymer membranes with optimized performance.

## 5. Conclusions 

This review article provided an overview of the employment of polymerized ILs in PEMFC applications. The review focused on the preparation of composite membranes utilizing the most commonly used polymers in PEM: Nafion, PVDF, PBI, SPEEK, and SPI, in addition to other polymers incorporated with ILs. A small section on the methods and techniques available for the synthesis of polymer/IL membranes was also presented. The addition of some carefully selected ILs to the polymer matrix improved the conductivity, thermal and mechanical stability, as well as the mechanical strength of pristine PEM. In terms of conductivity, PBI-based composite membranes revealed the highest conductivity and longest stability when compared to their polymer counterparts. For instance, conductivities of 371 and 309 mS/cm were recorded for PIL 2.5 mol% CL and PIL-PBI composite membranes. The same membranes exhibited a stable conductivity larger than 250 mS/cm and lasted for more than 40 days. With respect to the mechanical properties, it was concluded that protic ILs might produce a plasticizing effect, which causes a decline in the ionic domains’ dipolar interactions. A prominent method to overcome the issue is polymer cross-linking. In that aspect, SPEEK-based polymers exhibited the highest mechanical strength. For example, crosslinking glycerol or glycol with SPEEK to prepare TFAPA/SPEEK improved the membrane’s mechanical strength. The paper also highlighted the current challenges facing the employment of polymer/ILs in PEMFC and suggested various paths for the development of research on polymerized ILs and their application in PEMFC.

## Figures and Tables

**Table 1:** Summary of membranes classification.

Category	Structure
Perfluorinated Membranes	Fluorinated backbone with fluorocarbon side chain. Ex. Nafion
Partially Fluorinated Membrane	Fluorocarbon or Hydrocarbon base. Ex. PVDF
Nonfluorinated Membrane	Hydrocarbon or aromatic base. Ex. PBI
Acid–base Membranes	Integration of acid in an alkaline polymer base. Ex. H_3_PO_4_
Ionic Liquid Membranes	Formed between an organic cation and an organic/inorganic anion

**Table 2:** Conductivity of common protic IL in fuel cells.

Protic IL	Conductivity (mS/cm)	Temperature (°C)	Ref.
[dema][TfO]	43	120	[46]
[dema]HSO_4_	1.1	30	[47]
[dema][TFSA]	7.4	30	[47]
[MTBDH]TFSI	1.54	30	[48]
[bzlm][TFSI]	8.3	140	[42]
(Btmps)/HN(Tf)_2_	1	100	[49]
[C_2_H_3_N_3_]/[CH_3_SO_3_H]	149	200	[50]
[MIm][TFSI]	7.23	25	[51]
[MPz][TFSI]	12	90	[52]
ITSA	32.6	150	[53]
TFAPA	30	180	[54]
TEA-TF	31	130	[55]
TOA-TF	0.0303	25	[56]
[DMEIm]H_2_PO_4_	70	120	[57]
BG-BF_4_	180	180	[58]
[EMIm]HSO_4_	16	85	[59]
[morph][HCOO]	9.92	60	[60]
[Mmorph][HCOO]	16.77	60	[60]
[Emorph][HCOO]	12.17	60	[60]
[pyrr][NO_3_]	50.1	25	[61]
[pyrr]HSO_4_	5.8	25	[61]
[pyrr][HCOO]	32.9	25	[61]
[pyrr][CH_3_COO]	5.9	25	[61]
[pyrr][CF_3_COO]	16.4	25	[61]
[pyrr][C_7_H_15_COO]	0.8	25	[61]
[pyrr][TFSA]	39.6	130	[62]
[EMIM][TFSI]	5.4	150	[63]
[BMIm][BF_4_]	1.2	25	[64]

**Table 3:** Conductivity values of some Nafion-based membranes.

Proton Exchange Membrne	Proton Conductivity (mS/cm)	Temperature (°C)	Ref.
Nafion 117/imidazole-imidazolium	3–4	100	[72]
Nafion/[BMIM][TfO]	24	160	[74]
Nafion/[EIM][TfO]	5.5	160	[74]
Nafion/(TEA-PSHSO_4_)	159	80	[76]
Nafion/[MIMCM]Cl	6	130	[78]
Nafion/(SiO_2_)_3.67_(TEA)/(TEA-TF)_1.2_	4.7	105	[79]
Nafion/[C_3_OHMIM]BF_4_/SiO_2_ (50/10 wt%)	55	160	[80]
Nafion/[TMSPMIM]BF_4_/SiO_2_ (10 wt%)	375	90	[81]
Nafion/[DMBIM]H_2_PO_4_/GO (0.75/0.75/0.015 g)	6.1	110	[82]

**Table 4:** Conductivity values of some PVDF-based membranes.

Proton Exchange Membrane	Conductivity (mS/cm)	Temperature (°C)	Ref.
PVDF-HFP/EImTFSI (80 wt%)	10	25	[85]
PVDF-HFP/s-DFBP-HFDP/[EMIm](FH)_n_F)	34.7	130	[86]
HClO_4_SiO_2_/[dema][TfO]/PVDF-HFP	0.02 0.6	30 100	[88] [88]
PVDF/[N1114]^+^HSO_4_	10	140	[87]
60 wt% PAI/5 wt.% PVDF	7.5	150	[53]
PVDF-HFP/EImTfO	13.5	25	[93]

**Table 5:** Conductivity values of some PBI-based membranes.

Proton Exchange Membrane	Conductivity (mS/cm)	Temperature (°C)	Ref.
PBI/[HMI][TFSI]	54	200	[95]
[h-MIM] Ntf_2_/PBI	1.86	170	[96]
H_3_PO_4_/PMIH_2_PO_4_/PBI	2.0	150	[97]
PBI/[dema][TfO]	20.73	160	[98]
PBI/[BMIm]H_2_PO_4_	133	160	[99]
PBI/[MIm][TFSI]	100	160	[100]
PIL (crosslinker: 2.5 mol%)	371		[101]
PIL-PBI	309		[101]
PBI/IL/DVB (0.88/58.5/1%)	53.3	200	[102]
HPBI/IL/DVB (49.5/58.5/1%)	85	200	[103]
[PBI4N(ZrO_2_)_0.231_] (H_3_PO_4_)_13_	104	180	[104]
PBI/PA/ILGO	35	175	[105]
PBI/PA/PDC_3_	78	180	[106]
PBIOH-ILSi	106	170	[107]

**Table 6:** Conductivity values of some SPEEK-based membranes.

Proton Exchange Membrane	Conductivity (mS/cm)	Temperature (°C)	Ref.
SPEEK/[EMIm][DEP]	3	145	[108]
SPEEK/SiO_x_/[dema][TfO] (50 wt.%), SPEEK sulfonation degree: 66%	20	220	[109]
SPEEK/[dema][TfO]/Mmtdema, SPEEK sulfonation degree: 73%	78	70	[110]
SPEEK+[BMIm][BF_4_]/SiO_2_ (50/7.5 wt.%)	15	200	[111]
SPEEK/[dema][TfO] and 6 wt.% of ZrO_2_	660	70	[112]
SPEEK/[BMIm]BF_6_ (50%)/PA	30	160	[113]
(SPEEK/PU/SPEEK/BMiM)_100_/60%PA	103	160	[39]
6% SBA-15/SPEEK	10.2	140	[115]

**Table 7:** Conductivity values of some polymer-based membranes.

Proton Exchange Membrane	Conductivity (mS/cm)	Temperature (°C)	Ref.
ABPBI/SNR/[BMIm][TFSI]	10	80	[121]
SPAEKS/PWA	127	80	[122]
Poly (arylene ether)/[BMIm][BF_4_]	75	70	[123]
[BIm][DBP]/Matrimid^®^	20	115	[124]
MAT14PVP7/TEA-T	20	130	[55]
[ETFE-g-poly(4-VP)-SO_3_H] HSO_4_	259	90	[125]
[ETFE-g-P(1-Vim) _PrSO3H_]CF_3_SO_3_	138	95	[126]
[MIm][TfO]/PAM/PEG IPN (22.84 wt%)	10.37	150	[127]
[MIm][TfO]/PAM/PEG IPN (50 wt%)	17.02	150	[127]
[MIm][TfO]/PAA	19.4	200	[128]
[MIm][TfO]/PEG	40.4	200	[128]
[EIm][TFSI]/PEO	14	180	[129]
[MIm][TfO] and [APMIm][BR]-GO	14.8	160	[131]
PVA/PMA/SiO_2_/BMITFSI	0.83	60	[132]
PVA/PMA/SiO_2_/EMI-BF_4_	0.58	60	[132]
PVA-CA-EAN (1:0.05:0.4 molar ratio)	7.8	140	[133]
PPO/MeIM	67.9	30	[134]
ZrP/[EMIM] [ESO_4_	22.6	200	[139]
ZrP/[EMIM][SO_4_]/GLY/PTFE	70	200	[140]
TIB/QPPO	55	80	[142]
PANI/ZrP/IL (3.7 wt%)	20	25	[143]
CP/PTFE/[HMIM][C_4_N_3_^−^]	100	25	[40]
CP/PTFE/[HMIM][C_4_N_3_^−^]	3.14	200	[40]
SPPO	94	25	[144]
SPPO/N-methyl-2-pyrrolidone	11.6	25	[145]
SPAEKS/HPW-ILs@MIL-100	138	100	[147]
SPAEKKS	32	80	[148]

## Data Availability

Data will be available on request.

## Abbreviations

[2-FPy]TF:	2-fluoropyridinium triflate
[2-sea][TfO]:	2-Sulfoethylammonium trifluoromethanesulfonate
9FDA:	4,4′-(9-fluorenylidene) dianiline
ABPBI:	Poly(2,5-benzimidazole)
BG-BF_4_:	N-butylguanidinium tetrafluoroborate
[BIm][BEHP]:	1-n-butylimidazolium bis(2-ethylhexyl)phosphate
[BIm][DBP]:	1-n-butylimidazolium dibutylphosphate
BMIHSO_4_:	1-butyl-3-methylimidazolium hydrogen sulfate
[BMIM][DCA]:	1-butyl-3-methylimidazolium dicyanamide
[BMIm]H_2_PO_4_:	1-butyl-3-methylimidazolium dihydrogenphosphate
[BMIM][TFA]:	1-butyl-3-methylimidazolium triflate
[BMIM][TFA]:	1-butyl-3-methylimidazolium Trifluoroacetic
[BMIm][TfO]:	1-butyl-3-methylimidazolium trifluoromethanesulfonate
[BMIm][BF_4_]:	1-butyl-3-methylimidazolium tetrafluoroborate
[BMIm]PF_6_:	1-butyl-3-methylimidazolium hexafluorophosphate
(Btmps)/HN(Tf)_2_:	3-(1-butyl-1H-imidazol-3-ium-3-yl) propane-1- sulfonate 1,1,1-Trifluoro-N-(trifluoromethylsulfonyl) methanesulfoneamide
[bzlm][TFSI]:	Benzimidazolium bis(trifluoromethanesulphonyl)imide
[C_2_H_3_N_3_]/[CH_3_SO_3_H]:	1H-1,2,4-triazole/methanesulfonic acid
[C_2_mim][TFSI]:	Methylmethacrylatein1-ethyl-3-methylimida- zolium bis(trifluoromethanesulfonyl)imide
[C_3_OHmim]BF_4_:	1-(3-hydroxypropyl)-3-methylimidazolium tetrafluoroboride
CF_3_SO_3_:	Trifluoromethanesulfonate
CL:	Crosslinker
CP:	Calcium phosphate
[dema]HSO_4_:	Diethylmethylammonium hydrogensulfate
[dema][TfO]:	Diethylmethylammonium trifluoromethanesulfonate
[dema][TFSA]:	Diethylmethylammonium bis(trifluoromethylsulfonyl) amide
[DMBIm]H_2_PO_4_:	2,3 dimethyl-1-butylimidazolium dihydrogen phosphate
[DMEIm]H_2_PO_4_:	2,3-dimethyl-1-ethylimidazolium dihydrogenphosphate
[DEMET][TFSI]:	N,N-diethyl-N-methyl-N-(2-methoxyethyl) ammonium bis (trifluoromethanesulfonyl) imide
DSDA:	3,3′, 4,4′-diphenyl sulfone tetracarboxylic dianhydride
DTA^+^:	n-dodecyltrimethylammonium
DVB:	Divinylbenzene
[EIm][TfO]:	1-ethylimidazolium trifluoromethanesulfonate
[EIm][TFSI]:	N-ethylimidazolium bis(trifluoromethylsulfonyl)imide
[Emim]:	1-ethyl-3-methylimidazolium cation
[EMIM][AC]:	1-ethyl-3-methylimidazolium acetate
[EMIM][BF_4_]:	1-ethyl-3-methylimidazolium tetrafluoroborate
[EMIm][DEP]:	1-ethyl-3-methylimidazolium diethyl phosphate
[EMIM][ESO_4_]:	1-ethyl-3-methylimidazolium ethyl sulfate
[EMIm](FH)_n_F):	1-ethyl-3-methylimidazolium fluor hydrogenates
[EMIm]HSO_4_:	1-ethyl-3-methylimidazolium hydrogensulfate
[EMIM][OCSO_4_]:	1-ethyl-3-methylimidazolium octylsulfate
[EMIM][SO_4_]:	1-ethyl-3-methylimidazolium sulfate
[EMIM][TFSI]:	1-ethyl-3-methylimidazolium-bis (trifluoromethanesulfonyl) imid
[Emorph][HCOO]:	N-ethylmorpholinium formate
[Epdy]:	Ethyl pyridinium
ETFE:	poly(ethylene-co-tetrafluoroethylene)
ETFE-g-poly(4-VP):	Poly(4-VP) grafted ETFE films
ETFE-g-P(1-Vim):	Poly(1-vinyl imidazole) grafted onto ETFE film
ETS-10:	Microporous titanosilicate
H_3_PO_4_:	Phosphoric acid
H_2_SO_4_:	Sulfuric acid
HClO_4_SiO_2_:	Perchloric acid immobilized on nano-silica
[HMI][TFSI]:	1-H-3-methylimidazolium bis(trifluoromethanesulfonyl)imide
HPBI:	Hierarchical porous polybenzimidazole
HSO_3_-BBIm][TfO]:	1-butyl-3-(4-sulphobutyl)-imidazoliumtri-fluoromethanesulphonate
[HSO_3_-BBIm][TFSI]:	1-methyl-3-(4-sulfobutyl)-imidazolium bis (trifluoromethylsulfonyl)-imide
[HSO_3_-BMIm][TFSI]:	1-methyl-3-(4-sulphobutyl)-imidazolium bis(trifluoromethylsulphonyl)imide
IL: Ionic Liquid	
ImHSO_4_:	Imidazolium hydrogen sulfate
[ImVH][TfO]:	1-vinylimidazolium trifluoromethanesulfonate
[ImVH][TFSI]:	1-H-3-vinylimidazolium bis(trifluoromethanesulfonyl) imide
IPTS30:	IPTS grafted to PBIOH30 backbone
ITSA:	Isobutyramide trifluoromethanesulfonate
MAT14PVP7:	Interconnected porous support prepared from MAT14 and PVP7
[MIm]BF_4_:	n-methyl imidazolium tetrafluoroborate
[MImCM]Cl:	1-carboxylmethyl-3-methylimidazolium chloride
[MIm][DBP]:	1-n-methylimidazolium dibutylphosphate
[MIm][TfO]:	1-methylimidazolium trifluoromethanesulfonate
[MIm][TFSI]:	Methylimidazolium bis(trifluoromethanesulfonyl)imide
MIHSO_4_:	1-methylimidazolium hydrogen sulfate
[Mmorph][HCOO]:	N-methylmorpholinium formate
Mmtdema:	Modified montmorillonite clay with dema+ cation
[morph][HCOO]:	Morpholinium formate
[MPz][TFSI]:	1-methyl-pyrazole N,N-bis(trifluoromethane-sulfonyl)imide
[MTBDH]TFSI:	7-methyl-1,5,7-triazabicyclo[4.4.0]dec-5-ene bis(trifluoromethylsulfonyl)imide
[N1114]^+^:	Trimethylethyl amide cation
[N1114]^+^HSO_4_:	Trimethylethyl amide hydrogensulfate
OCP:	Open circuit potemtial
PA:	Polyamide
PAA:	Poly(acrylic acid)
PEG:	poly(ethylene glycol)
PAI:	Polyamide imide
PAMAM:	Polyamidoamine
PAM/PEG IPN:	Polyacrylamide/polyethylene glycol interpenetrated
PANI:	Polyaniline
pBABTS:	1,4-bis(4-aminophenoxy-2-sulfonic acid) benzenesulfonic acid
PBI:	Polybenzimidazole
PBI-O-Ph:	Polybenzimidazole derivative with benzofuranone moieties
PDC_3_:	1,3-di(3-methylimidazolium) propane bis (trifluoromethylsulfonyl) imide
PEM:	Proton Exchange Membrane
PEMFC:	Proton Exchange Membrane Fuel Cell
PMC_6_:	1-hexyl-3-methylimidazolium bis (trifluoromethanesulfonyl) imide
PMIH_2_PO_4_:	1-propyl-3-methylimidazolium dihydrogen phosphate
PPO:	Poly(phenylene oxide)
PVA:	Plovinyl alcohol
PVDF:	Polyvinylidene fluoride
PVDF-HFP:	Poly(vinylidene fluoride-co-hexafluoropropene)
PWA:	1-ethyl-3-methyl imidazolium phosphotungstate
[pyrr][C_7_H_15_COO]:	Pyrrolidinium octanoate
[pyrr][CF_3_COO]:	Pyrrolidinium trifluoroacete
[pyrr][CH_3_COO]:	Pyrrolidinium acetate
[pyrr][HCOO]:	Pyrrolidinium formate
[pyrr]HSO_4_:	Pyrrolidinium hydrogensulfate
[pyrr][NO_3_]:	Pyrrolidinium nitrate
[pyrr][TFSA]:	Pyrrolidinium bis(trifluoromethanesulfonyl)amide
QPPO:	Poly (2,6-dimethyl phenylene oxide
RTIL:	Room temperature ionic liquid
SiO_2_:	Silicon oxide
SPAEKKS:	Sulfonated poly(arylene ether ketone ketone sulfone)
SPAEKS:	Sulfonated poly(arylene ether ketone sulfone)
SPEEK:	Sulfonated poly (ether ether ketone)
SPI:	Sulfonated polyimide
SPPO:	Sulfonated Polyphenylene Oxide
SPS:	Sulfonated polystyrene
TEA:	Triethylammonium
TEAH^+^:	Triethylammonium cation
TEA-TF:	Triethylammonium-triflate
TFA^−^:	Triflate anion
TFAPA:	Trifluoroacetic propylamine
TIB:	1,3,5-tris(1-imidazolyl)benzene
[TMPA][TFSI]:	N,N,N-trimethyl-N-propylammonium bis (trifluoromethanesulfonyl) imide
TMS:	Triethylammonium methanesulphonate
[TMSPMIm]BF_4_:	1-(3-trimethoxysilylpropyl)-2-methylimidazolium tetrafluoroborate
TOA-TF:	Trioctylammonium triflate
TPFBu:	Triethylammonium perfluorobutanesulphonate
ZrO_2_:	Zirconium dioxide
ZrP:	Zirconium phosphate
